# *Dhat* syndrome in relation to demographic characteristics

**DOI:** 10.4103/0019-5545.46077

**Published:** 2005

**Authors:** Nashi Khan

**Affiliations:** * Chartered Clinical Psychologist Punjab Institute of Mental Health, PO Box 3222, Gulberg, Lahore 54660, Pakistan

**Keywords:** *Dhat* syndrome, health professionals

## Abstract

**Background:**

The area of men's sexual health has been poorly understood, particularly with reference to South Asian cultures. The belief that losing semen is detrimental to health is a concept common to both Oriental and Occidental thinking.

**Aim:**

To estimate the number of patients with the *dhat* syndrome consulting different professionals in Lahore and to examine their demographic characteristics.

**Method:**

Seventy health professionals of various types were approached and asked to fill in a daily record form for all patients reporting at their clinics for a period of 1 month.

**Results:**

A total of 1777 patients were reported to attend the outpatient clinics of health professionals of various types over a period of 1 month. The majority of patients consulted *hakims* for professional help. Most of the patients were single, with a mean age of 24 years and had a monthly income of less than Rs 3000.

**Conclusion:**

These data have important implications for patients, professionals and researchers. The alarming number of people consulting various professionals to seek help for anxiety due to semen loss highlights the fact that research into and interventions for this neglected area of men's sexual health are urgently required.

## INTRODUCTION

The entire area of men's sexual health has been poorly understood, particularly in South Asian cultures. There are some contrasts and similarities in the understanding of issues relating to sexual health in these cultures among health professionals.

The word *dhat* is derived from the Sanskrit word *dhatu*, meaning metal and also elixir or a constituent of the body. It was first described in western psychiatric texts by Wig.^1^ The anxiety due to semen loss is well described in Indian historical writings. The syndrome is not confined to India; it is widespread in all communities of the Indian subcontinent.^2^–^4^

The belief that losing semen is detrimental to health and, conversely, that semen conservation is beneficial to mental and physical health is a concept common to both Oriental and Occidental thinking.^5^ Money *et al*. argued that the doctrine of semen conservation was incorporated into eighteenthcentury European medical theory by a Swiss physician, Tissot.^6^ His treatise on the diseases produced by Onanism (masturbation) was first published in 1758. The basic tenet of Tissot's theory was that debility, disease and death are the outcome of degeneracy induced by semen loss caused by masturbation and prostitution.^6^,^7^ In nineteenth-century England, Maudley held the view that semen loss, especially through masturbation, results in mental illness.^8^

According to Indian tradition (writings in the Upanishads), the term *virya* stands for both vigour and semen^9^ and is considered the source of physical and spiritual strength. In Indian culture, the loss of *virya* through any sexual act or imagery (including masturbation, *swapnadosh* [wet dreams]) is considered both physically and spiritually harmful.^10^ Thus, it can be inferred that in various cultures of the world, semen loss has been a concern and is believed to weaken the body and cause disease.

The ancient doctrine of semen conservation has a long philosophical history in Europe and Asia. In Sanskrit, semen is equated with *sukra*, the life force.^6^ In the Indian Ayurvedic system of medicine, *sukra* is considered essential for healthy functioning of the body. In Ayurvedic medicine, it is believed that loss of semen in any form leads to depletion of physical and mental energy.^11^ However, Mumford suggests that the complaint of *dhat* is not confined to Hindus, but is widespread among all communities of the Indian subcontinent.^12^ It has been reported among Sikhs in Punjab, Buddhists in Sri Lanka and Muslims in Pakistan.^12^ Several empirical studies have been conducted on the *dhat* syndrome in India.^13^,^14^

Singh studied 50 men with potency disorders attending a psychiatric clinic in Patiala. Among the total sample, 62% complained of *dhat* as a major symptom and the majority of patients reported various somatic symptoms.^2^ De Silva and Dissanayake studied semen loss among Sri Lankan males and their study population was also drawn from a psychiatric clinic.^4^ It was found that all patients shared a common set of beliefs about seminal fluid and its loss. The main source of these beliefs was the Ayurvedic medical tradition.

Bhatia and Malik studied symptom manifestation in a sample of men with psychosexual disorders attending a psychiatric clinic in Delhi.^14^ Of the 144 patients, 93 were identified as having the *dhat* syndrome. The chief complaints were weakness, fatigue, palpitations and poor sleep. Impotence and premature ejaculation were also frequently reported by these patients. Semen loss in the urine was very common among men presenting with psychosexual problems. Bhatia and Malik claim that their study supported the diagnosis of the *dhat* syndrome in the Indian culture.^14^

Mumford studied 394 men attending general medical clinics in Pakistan.^12^ The complaint of *dhat* was reported by 30% of the patients and it was strongly associated with erectile difficulty, depressed mood and symptoms of fatigue. Mumford demonstrated that in Pakistan, the complaint of *dhat* is also common in patients with general medical conditions.

Research on the *dhat* syndrome is scarce and there is little evidence to suggest that the syndrome is more common among men of lower socioeconomic status, those from rural areas, those belonging to families with conservative attitudes and those who have married recently.^13^,^15^

The cultural domain of men's sexual health in Pakistan can be understood by the general cover term which describes it as *mardana kamzori*. It is the most common term used to describe male sexual health problems in Urdu (the national language of Pakistan). *Mardana kamzori* is a generic term that attempts to explain problems related to the sexual functioning of men such as impotence and premature ejaculation, as well as concerns generated by local myths, e.g. masturbation, thinning of the semen and wet dreams. Problems related to the sexual health of men are highlighted in an exaggerated manner in newspaper advertisements, pamphlets and advertisements displayed on the walls of buildings under the heading *mardana kamzori ka ilaaj*.

In Pakistan, the *dhat* syndrome has remained unresearched. This may be due to the social taboos attached to the area of sexuality. Although anxiety caused by semen loss seems to be a major concern of males visiting different health clinics for sexual dysfunction, this problem has been completely ignored by professionals and researchers in Pakistan. In a male-dominated society such as that in Pakistan, it is commonly believed that a man's sexual strength is the best way to prove his manhood and superiority. Research is needed to estimate the number of those suffering from, as well as implications of, the syndrome. Sex is a taboo subject in Pakistan and any problem pertaining to it is ignored by researchers. The present study was a pioneering one that focused on estimating the number of patients with the *dhat* syndrome reporting at outpatient clinics of various professionals in Lahore. The study also examined the demographic characteristics of patients with the *dhat* syndrome.

It was hypothesized that the majority of patients with the *dhat* syndrome reporting at different outpatient clinics would be young, single, less educated and from a low socioeconomic class.

## METHODS

The focus of the study was to obtain the demographic characteristics of patients with the *dhat* syndrome.

Seventy health professionals—*hakims*, homeopaths, general practitioners, psychiatrists, psychologists, urologists and infertility specialists—in Lahore were approached for data collection. They were assured by the researcher that the information obtained would be kept confidential and would be used only for research purposes. Fifty-eight of them (83%) returned the completed record forms.

The health professionals were provided a list of ICD-10 criteria of the *dhat* syndrome to diagnose patients.^16^ Record forms seeking information about demographic characteristics such as age, marital status, education, time since onset of the problem were given to the professionals. Each health professional was required to complete the record forms for cases reporting daily over a period of 1 month.

## RESULTS

The total number of patients with the *dhat* syndrome reported by different health professionals was 1777. The data indicate that there is a significant difference in the number of cases consulting various types of professionals (χ^2^ = 2458, df = 6, p<0.001). The highest percentage of patients visited *hakims* (50%). The least number of cases was reported by urologists (0.3%, Table 1).

**Table 1:** Patients with the *dhat* syndrome visiting different health professionals for treatment (*n*=1777)

Health professional	Number visited	χ^2^ value
*Hakim*	883 (49.7)	2458*
Homeopath	419 (23.6)	
Physician	330 (18.6)	
Psychiatrist	28 (1.6)	
Psychologist	24 (0.7)	
Urologist	5 (0.3)	
Infertility specialist	100 (5.6)	

* p<0.001 Values in parentheses are percentages.

The patients ranged in age from 12 to 65 years with a mean age of 24 years (SD 8.5). The patients differed significantly in their demographic characteristics such as marital status (χ^2^ = 2628, df = 3, p<0.001), education level (χ^2^ = 236.8, df = 1, p<0.001) and socioeconomic status (χ^2^ = 663, df = 2, p<0.001). The majority of patients were single (75%), educated up to the matriculation level (class X) (67%) and had a monthly income of less than Rs 3000 (56%, Table 2).

**Table 2:** Demographic characteristics of the patients (*n*=1777)

Characteristic	Number	χ^2^ value
Marital status		
Single	1332 (75)	2628.3*
Married	387 (21.8)	
Widowed	23 (1.3)	
Divorced	14 (0.8)	
Data not mentioned	21 (1.2)	
Educational level		
Up to matriculation (class X)	1191 (67)	236.87*
Above matriculation	549 (31)	
Data not mentioned	36 (2)	
Monthly income		
< Rs 3000	989 (55.7)	663.6*
Rs 3000–12,000	536 (30.2)	
Rs 12,000–50,000	133 (7.5)	
Data not mentioned	119 (6.7)	

* p<0.001 Values in parentheses are percentages.

Sixty per cent of the patients were concerned about their semen loss for the past 6 months. Only 14% of the patients had the *dhat* syndrome for more than 2 years.

## DISCUSSION

The present study examined the demographic characteristics of patients with the *dhat* syndrome consulting various types of health professionals in Lahore. The results show that 73% of the patients consulted *hakims* and homeopaths. A similar finding was reported by De Silva and Dissanayake, who found that the majority of their sample sought the help of ayurvedic and homeopathic practitioners.^4^ One possible explanation for *hakims* and homeopaths being frequently visited by such patients could be that these two professionals flamboyantly advertise this syndrome as a major health concern, which would convey empathy and understanding from such practitioners for those who suffer from this problem. They propagate the idea that semen is a precious body fluid and its loss leads to many physical and mental problems, especially impotency, resulting in increased clientele of these professionals. Other professionals do not propagate semen loss as a major concern and patients may visit them for other medical reasons.

The syndrome is most prevalent in young and single men. These findings are consistent with existing research done in other cultures.^2^,^11^ The findings are also in agreement with those of De Silva and Dissanayake, and Bhatia and Malik, who found a preponderance of single males among their study population.^4^,^14^ This preponderance of unmarried males could be due to the fact that they are worried about the implications of semen loss for their future marital life and therefore seek treatment earlier. Consequently, there may be a delay in marriage because of the prevailing belief that they have to rid themselves of this affliction if they are to enjoy a normal, healthy, sexual life.

In the present study, 60% of patients had been anxious about their semen loss for the past 6 months; only 14% reported a duration of illness for more than 2 years. This shows that men with the *dhat* syndrome are concerned about their problem and its perceived consequences, and would therefore readily consult health practitioners for its relief.

Another important finding was that the majority of the patients were educated up to class X and only 31% had a higher educational background. The findings support the research hypothesis and are in agreement with those of Singh. In his study also, the majority of patients were educated up to matriculation.^2^ This indicates that level of education plays a key role in this syndrome and that it is more prevalent in less educated people.

The majority of patients were from a low socioeconomic status, which would consequently lower their standard of living. This finding is in agreement with that of Verma *et al*., who reported that most patients in their study had a low-to-medium standard of living.^10^ It can be argued that the patients' education level was also low, which would be related to their socioeconomic status as well as their standard of living.

In conclusion, the *dhat* syndrome seems to be one of the major concerns of young, single, less educated men from a low socioeconomic class. The findings from the present study highlight the importance of further research to investigate the likely psychological impact of the syndrome on those who suffer from it. Moreover, the huge number of patients consulting professionals during a short period of time highlights the importance of research to explore different aspects of men's sexual health in our society, which is primarily conservative and uptight in relation to sexual issues. The present study can become an important milestone in identifying the psychological and physical implications of this syndrome and taking effective measures to deal with it.
